# Mediating role of arsenic in the relationship between diet and pregnancy outcomes: prospective birth cohort in Bangladesh

**DOI:** 10.1186/s12940-019-0450-1

**Published:** 2019-02-06

**Authors:** Pi-I D. Lin, Sabri Bromage, Md. Golam Mostofa, Mohammad Rahman, Joseph Allen, Emily Oken, Molly L. Kile, David C. Christiani

**Affiliations:** 1 Department of Environmental Health, Harvard T.H. Chan School of Public Health, 677 Huntington Ave, Boston, MA 02115 USA; 2 Department of Nutrition, Harvard T.H. Chan School of Public Health, 677 Huntington Ave, Boston, MA 02115 USA; 3 Department of Environmental Research, Dhaka Community Hospital, 190/1 Wireless Railgate Bara Moghbazar, Dhaka, 1217 Bangladesh; 4 Department of Population Medicine, Harvard Medical School and Harvard Pilgrim Health Care Institute, 401 Park Drive, Suite 401 East, Boston, MA 02215 USA; 5 College of Public Health and Human Science, Oregon State University, 160 SW 26th St, Corvallis, OR 97331 USA; 6 Research Center for Environmental Medicine, Kaohsiung Medical University, No. 100 Shiquan 1st Road, Sanmin District, Kaohsiung City, Taiwan 807 Taiwan

**Keywords:** Arsenic exposure, gestational age at birth, gestational weight gain, birth weight, Fetal growth, Preterm birth, Mediation analysis, Maternal diet, Food frequency questionnaire, Rural Bangladesh

## Abstract

**Background:**

Epidemiological evidence suggests that arsenic (As) exposure during pregnancy may reduce infant birth weight. One significant source of As exposure is diet; thus, As may indirectly affect infant growth by mediating the effect of maternal diet on birth weight (BW). This study evaluated the potential mediating effect of As in the relationship between maternal diet and BW, gestational age (GA), and gestational weight gain (GWG).

**Method:**

The study used a prospective birth cohort in Bangladesh that captured the dietary habits of 1057 pregnant women through validated semi-quantitative food frequency questionnaires. We applied a causal mediation model with counterfactual approach and performed analyses with and without adjustment for total energy intake. Other potential confounders captured by self-report questionnaire were exposure to secondhand tobacco smoke, betel nut chewing, maternal age, education level, household income level, physical activity level during pregnancy, and daily hours spent cooking over open fire.

**Result:**

No association was found between maternal toenail As and BW. Higher absolute and energy-adjusted protein, fat and fiber intakes were associated with higher toenail As and lower GA and GWG, while higher absolute and energy-adjusted carbohydrate intake was associated with lower toenail As and greater GA and GWG. Mediation analysis showed significant natural indirect effects by toenail As in the relationships between absolute fat, carbohydrate and fiber intake with GA. Specifically, 3% (95% CI: 1–6%) of the association between carbohydrate intake and GA was mediated by change in toenail As, 6% (95% CI: 1–9%) for absolute fat intake and 10% (95% CI: 4–13%) for absolute fiber intake. After adjusting for total energy, no significant mediating effect was observed, suggesting the mediating effect might be due to measurement error or that absolute amount of As exposure rather than the amount in relationship to total energy intake was a more important factor to consider when understanding the negative implication of As on fetal growth.

**Conclusion:**

The mediating effect of As in the relationship between maternal diet and birth outcome was small and might be due to measurement error.

**Electronic supplementary material:**

The online version of this article (10.1186/s12940-019-0450-1) contains supplementary material, which is available to authorized users.

## Background

Arsenic (As), a ubiquitous naturally occurring metalloid, is designated by the World Health Organization (WHO) as a major chemical toxicant impacting more than 140 million people globally [1, 2]. Many countries report elevated As concentrations [3] in ground water; Bangladesh reports the most significant problem [4]. In addition to contaminated water, As exposure can occur through consumption of contaminated food. Yet, compared to As exposure from water, dietary sources of As have received relatively less attention. In areas with lower As level in the groundwater, dietary intake has been identified as the major source of As exposure [5, 6]. A survey of food composites in Canada reported high mean concentrations of As in fish (1662 ng/g), meat and poultry (24.3 ng/g); baked good and cereals (24.5 ng/g); and fat and oils (19.0 ng/g) [7]. Agricultural products can accumulate As from contaminated soil, water, and pesticides [8–10], while As in livestock products may derive from feed, feed supplements and foraged grass and plants [8–13]. The daily exposure to inorganic As is estimated at 1.7–3.0 μg/kg body weight/day for adults in Bangladesh [14], which is much higher than levels in most countries in Europe and North America [15].

Arsenic is a suspected reproductive toxicant because it readily crosses the placental barrier from mother to fetus [16]. Indeed, high levels of As in groundwater (≥ 50 μg/L) are associated with greater risk of spontaneous abortion, stillbirth, and moderate risk of neonatal mortality and infant mortality [17]. Further, low to moderate As exposure levels are associated with lower birth weight (BW) [17–21], gestational age (GA) [21, 22], birth length, and chest and head circumferences [20]. Active surveillance from 2004 to 2007 incidated that 21% of newborns in Bangladesh were preterm (< 37 weeks of gestation), and 55.3% had low BW (LBW, < 2500 g), representing the highest incidence of LBW babies [23]. Our team established a prospective birth cohort (2008–2011) to investigate the effect of chronic As exposure on the health of pregnant women and their offspring in an area of Bangladesh with a moderate range of As level in the drinking water. We found that lower BW is associated with elevated maternal total As exposure in a dose-dependent manner [22, 24], underscoring the risk between BW and maternal As.

The interaction of environmental exposure to toxicants and maternal nutrient intake is complex and not well understood [25]. For example, fish consumption provides both beneficial nutrients, such as docosahexaenoic acid, an omega-3 fatty acid required for brain and retinal development, and methylmercury, a toxicant that promotes adverse neurodevelopment [26–28]. The interaction between As exposure and nutrients has mainly focused on the role of folate on As metabolism, and most prior studies were conducted on undernourished populations [29, 30]. Thus, the interaction of As exposure with maternal diet and its effects on birth outcomes remains unclear. Evidence from our cohort suggests that maternal consumption of certain food dishes, including vegetables, fish and meat, are associated with higher toenail As levels among pregnant women [31]. We hypothesize that part of the adverse effect of As exposure on BW might be mediated through cumulative exposure to As. Previous studies were unable to examine this potential mediation using linear regression methods. To separate the direct effect of maternal diet on BW and the indirect effect mediated via increasing As exposure, we employed a causal mediation model [32] to investigate levels of mediation by total As exposure, as measured by maternal toenail As, in the relationship between maternal diet and BW. Our previous analysis using structural equation modeling suggested that the effect of As on BW was mediated via decreased GA at birth and reduced gestational weight gain (GWG) [22]; thus, we also explored these two birth outcomes while testing the interaction of maternal diet with toenail As.

## Methods

### Study population and data collection

**Fig. 1:**
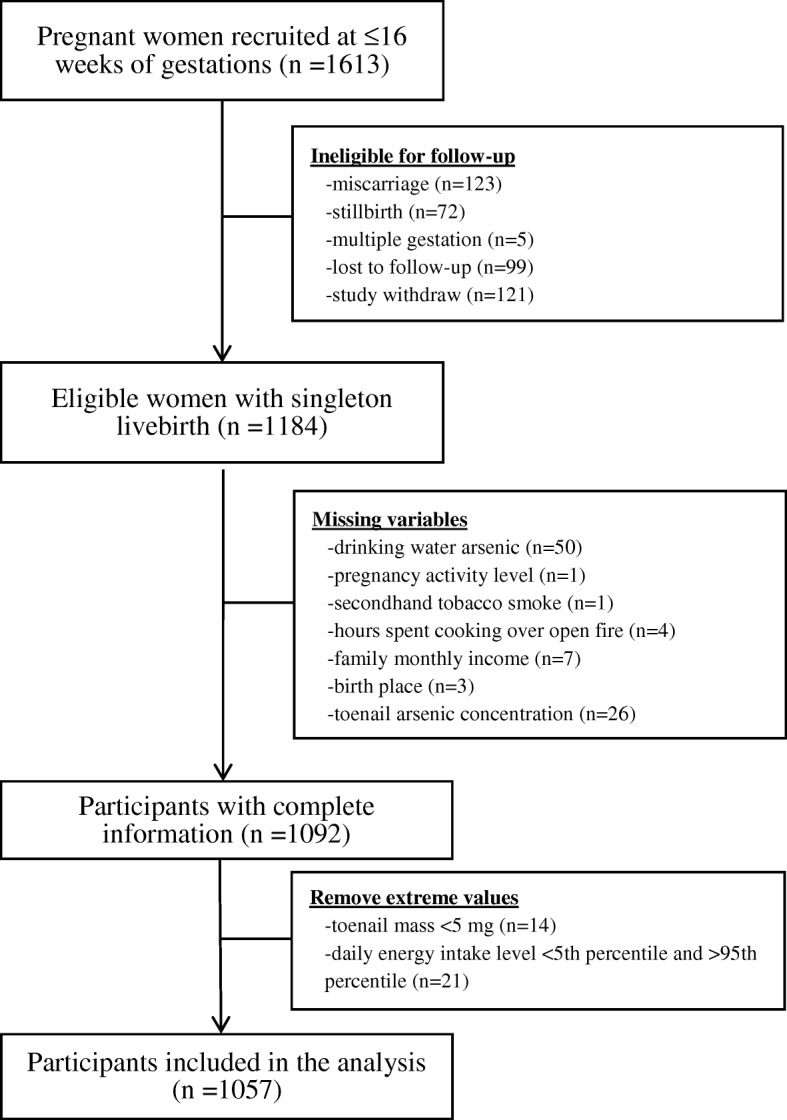
Study flowchart

We included 1184 pregnant women who had a singleton livebirth from a prospective birth cohort (2008–2011) in Sirajdikhan and Pabna Sadar Upazilas of Bangladesh, where the As level in groundwater was moderate over a wide range. The cohort has been detailed elsewhere [22, 24, 33, 34]. Participants provided written informed consent. The study protocol was approved by the institutional review boards of Dhaka Community Hospital (DCH) and Harvard T.H. Chan School of Public Health (IRB number P11351, approved February 2008). After excluding pregnant women with missing variables (*n* = 92), toenail mass < 5 mg (*n* = 14), and extreme daily energy intake <5th and > 95th percentile (*n* = 21), the sample size was 1057 (Fig. 1).

### Analysis of as exposure

Toenail As concentration is a reliable biomarker for long-term cumulative As exposure [35, 36]. We used maternal toenails collected at one month postpartum to estimate the total As exposure level accumulated throughout pregnancy [34]. Toenail samples were acid-digested by microwave using the method described in Chen et al. [37], and total As concentration in the digested samples was analyzed by inductively coupled plasma mass spectrometry (ICP-MS; Perkin Elmer, Shelton, CT, USA) [22, 33, 34]. All reported analytical values were blank-corrected. Inter-batch differences in instrument performance were accounted for by multiplying the analytical values by the inverse of the batch-specific percentage recovery in the CRM (mean percentage recovery for As was 76%). The mean limit of detection (LOD) for toenail As was 0.04 μg/g, and the relative standard deviation (SD) was 6.1%.

We examined As concentration of each participant’s primary drinking source. Water samples were collected at one month postpartum and analyzed by ICP-MS using the US EPA method 200.8. The instruments had recoveries of 98 to 107% when tested with spiked laboratory control (ICP, Analytical Mixture 12 Solution A, High Purity Standard, Charleston, SC, USA). We assigned half of the LOD to water samples that were below the LODs (*n* = 246).

### Assessment of energy and macronutrient intakes

We used a locally validated dish-based semi-quantitative food frequency questionnaire (FFQ) [38] to collect dietary information. The FFQ showed good validity for measuring total energy (Spearman r = 0.35, *p* < 0.01), protein (Spearman r = 0.46, p < 0.01), fat (Spearman r = 0.45, *p* < 0.01), carbohydrate (Spearman r = 0.50, *p* < 0.01) and fiber (Spearman r = 0.43, *p* < 0.01) when compared with food diaries in a previous study [38]. Health care workers trained to administer the FFQ interviewed pregnant women one month postpartum to recall their dietary habits for the preceding 12 months. Methods to calculate total energy and nutrient intakes were described previously [31, 38].

### Assessment of birth outcomes and other covariates

All women received ultrasound examination at the time of enrollment and during the 2nd trimester to estimate GA. GA was recorded by trained health care workers at the time of delivery. Maternal prenatal body weights were measured at monthly follow-ups by health care workers using a calibrated scale and GWG was calculated as a function of gram/week using the estimated slope of the linear regression model between monthly weight and gestational week from the 14th week to the last available weight measurement before delivery. BW was measured at the location of delivery (45% measured at a hospital or clinic and 55% at participant’s home) by a trained health care worker, using a pediatric scale that was calibrated before each use and rounded to the nearest 10 g. Newborn sex, birth delivery location, and birth delivery type were recorded at the time of delivery by the research staff using a standardized reporting form. Other covariates used in the analysis were captured by self-report questionnaire; these included exposure to secondhand tobacco smoke, betel nut chewing, maternal age, education level, household income level, physical activity level during pregnancy, and daily hours spent cooking over open fire.

### Statistical analysis

Continuous variables were assessed for normality using the Shapiro-Wilk test statistic. Toenail and water As concentrations were right skewed and therefore transformed to their natural logarithm (ln) to improve normality of the residual in the regression model. Descriptive statistics were computed for all variables. *T*-test or analysis of variance was used to compare mean birth outcomes across categories of all covariates in bivariate analysis.

**Fig. 2:**
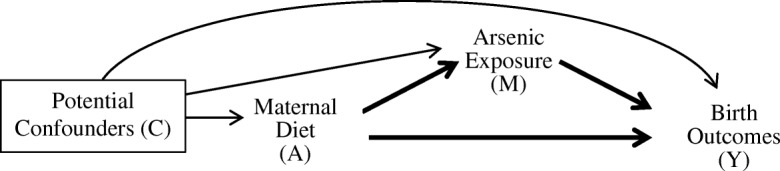
Simple conceptual model for mediation analysis in the context of the present study

We summarize our conceptual model in Fig. 2. The exposures (A) used in the analysis were measures of maternal diet, including total energy (kcal/day), protein (gram/day), fat (gram/day), carbohydrate (gram/day) and fiber (gram/day) intakes, and the outcomes (Y) were birth outcomes, including BW (gram), GA (week), and GWG (g/week). Ln-transformed toenail As [ln(μg/g)] was tested as the mediator (M). Simple and multiple linear regression models were used to evaluate the linear relationship between (1) A-Y, (2) M-Y, and (3) A-M, respectively. Three models were fitted for each of the relationships, including (1) a crude model, (2) an energy-adjusted model where maternal diets and toenail As were adjusted for total energy intake using the residual method [39], and (3) a fully-adjusted model, where additional potential confounders (C) were added to the energy-adjusted model. To ensure comparability among effect estimates, increments of 1 SD were used for energy and macronutrient intakes. Potential confounders (C) controlled in the multiple linear regressions included body mass index (BMI) at the time of enrollment, exposure to secondhand tobacco smoke, betel nut chewing, maternal age, education level, household income level, newborn sex, birth delivery location, birth delivery type, physical activity level during pregnancy, daily hours spent cooking over open fire. None of the pregnant women smoked; thus, maternal smoking was not a possible confounder.

We tested the mediation effect of toenail As in the association of maternal diet and birth outcomes by employing a mediation analysis with counterfactual approach (Fig. 2), using the SAS macro developed by Valeri and VanderWeele [32]. The causal mediation analysis has four assumptions, including (1) no unmeasured exposure-outcome confounder conditioned on C, (2) no unmeasured mediator-outcome confounder conditioned on A and C, (3) no unmeasured exposure-mediator confounder conditioned on C, and (4) no mediator-outcome confounder affected by exposure. When these assumptions hold, the natural direct effect (NDE) represents the effect on birth outcomes if maternal diet were changed from the sample mean to mean minus 1 SD, while keeping the toenail As level for each individual at that for intake level equal to sample mean minus 1 SD. The natural indirect effect (NIE) represents the estimated effect on birth outcomes when controlling maternal diet at the sample mean while changing the toenail As level from the level it would have been at maternal diet equal to sample mean minus 1 SD, to that for intake level equal to sample mean. The proportion of mediation by toenail As was calculated as the ratio of NIE to total effect (TE), which is the overall effect of exposure on outcome. Two sets of mediation analysis were performed, one using maternal diet and toenail As not adjusted for energy intake, and the other using energy-adjusted maternal diet and toenail As concentration, both models adjusted for potential confounders. The unadjusted model evaluated the effect of absolute exposure, while the energy-adjusted analysis evaluated the effect of proportional composition of exposure level using isocaloric comparisons. We tested for exposure-mediator interaction by running mediation analyses both with and without interaction. An interaction was present when a significant difference was observed in the effect estimates comparing models with and without interaction. Due to strong correlation of water arsenic concentration and toenail arsenic concentration, we did not adjust for water arsenic in the model; instead, we performed sensitivity analyses by stratifying by variables that may modify the effect of maternal diet and As exposure on birth outcome, including drinking water As level (≥50 μg/L and < 50 μg/L) [38]. We also performed sensitivity analysis stratified by BMI category at enrollment. In all tests, a *p*-value of less than 0.05 was considered statistically significant, and all tests were two-tailed. Statistical analyses were performed using SAS Software version 9.3 (SAS Institute, Cary, NC, USA).

## Results

### Characteristics of study population

The average GA in this population was 38.0 weeks (SD: 1.8 weeks; interquartile range (IQR): 31, 42 weeks), the average GWG was 360 g/week (SD: 120 g/week; IQR: 40, 780 g/week), and the average BW was 2843 g (SD: 397 g; IQR: 2610–3100 g). The median toenail As concentration was 1.21 μg/g (IQR: 0.65, 2.96 μg/g) (Table 1). Overall, women with Cesarean births, who gave birth in hospitals, and who came from a family with higher household income had higher GA, GWG and BW. Women with higher drinking water As, higher toenail As and exposure to secondhand smoke had lower GA, GWG and BW. Mean difference in GA, GWG and BW all decreased across increasing quartiles of protein, fiber and fat intakes, and in contrast, increased across quartiles of carbohydrate intake (all p-for-trend < 0.001) (Table 2).

**Table 1:** Characteristics of selected participants (*n* = 1057)

Characteristic	N (%)	GA (SD) in week	*P*-value	GWG (SD) in g/week	P-value	BW (SD) in kg	*p*-value
Maternal age (y)							
18–20	415 (39.3)	38.06 (1.77)	0.298	0.38 (0.13)	< 0.001	2833 (359)	0.662
21–25	393 (37.2)	37.89 (1.83)		0.35 (0.12)		2858 (425)	
26–41	249 (23.6)	38.08 (1.84)		0.33 (0.12)		2840 (409)	
BMI at enrollment (kg/m^2^)							
< 18.5	296 (28.0)	37.97 (1.77)	0.309	0.38 (0.13)	< 0.001	2792 (402)	< 0.001
18.5–25	663 (62.7)	37.97 (1.86)		0.36 (0.13)		2841 (379)	
25–30	88 (8.3)	38.35 (1.55)		0.31 (0.09)		3018 (463)	
> 30	10 (0.9)	37.90 (1.52)		0.34 (0.10)		2997 (284)	
Infant sex							
Male	536 (50.7)	38.02 (1.81)	0.773	0.35 (0.12)	0.214	2884 (370)	0.001
Female	521 (49.3)	37.99 (1.81)		0.36 (0.13)		2805 (417)	
Birth type							
Vaginal	692 (65.5)	37.78 (1.90)	< 0.001	0.35 (0.13)	0.001	2783 (401)	< 0.001
Cesarean	365 (34.5)	38.42 (1.55)		0.37 (0.12)		2959 (362)	
Birth location							
Home	581 (55.0)	37.75 (1.94)	< 0.001	0.35 (0.12)	0.002	2749 (389)	< 0.001
Clinic	69 (6.5)	37.81 (1.90)		0.35 (0.10)		2996 (360)	
Hospital	407 (38.5)	38.40 (1.51)		0.37 (0.13)		2964 (377)	
Drinking water arsenic (μg/L)							
< 50	833 (78.8)	38.24 (1.73)	< 0.001	0.36 (0.13)	< 0.001	2857 (376)	0.031
≥ 50	224 (21.2)	37.12 (1.82)		0.32 (0.11)		2793 (463)	
Toenail arsenic (μg/g)							
0.04–0.64	270 (25.5)	38.55 (1.57)	< 0.001	0.38 (0.14)	0.002	2881 (319)	0.176
0.65–1.20	259 (24.5)	37.97 (1.77)		0.36 (0.12)		2847 (375)	
1.21–2.95	263 (24.9)	37.82 (1.92)		0.35 (0.12)		2805 (450)	
2.96–46.58	265 (25.1)	37.66 (1.85)		0.34 (0.12)		2842 (428)	
Secondhand tobacco smoke							
No	613 (58.0)	38.05 (1.81)	0.298	0.36 (0.13)	0.010	2874 (393)	0.003
Yes	444 (42.0)	37.93 (1.82)		0.35 (0.12)		3801 (398)	
Betel nut chewing							
No	1046 (99.0)	38.00 (1.81)	0.244	0.36 (0.12)	0.642	2845 (397)	0.254
Yes	11 (1.0)	38.64 (1.75)		0.37 (0.13)		2708 (321)	
Maternal education							
Secondary	562 (53.2)	37.84 (1.79)	< 0.001	0.36 (0.12)	0.025	2729 (381)	0.001
Primary	342 (32.4)	38.37 (1.73)		0.36 (0.13)		2862 (380)	
Illiterate	153 (14.5)	37.78 (1.92)		0.34 (0.12)		2864 (405)	
Household monthly income (USD)							
< 36	180 (17.0)	37.69 (1.87)	< 0.001	0.37 (0.13)	< 0.001	2804 (402)	0.001
37–49	287 (27.2)	37.78 (1.89)		0.35 (0.11)		2837 (393)	
50–61	308 (29.1)	38.12 (1.77)		0.36 (0.13)		2807 (423)	
62–74	157 (14.9)	38.23 (1.72)		0.37 (0.13)		2877 (358)	
> 75	125 (11.8)	38.39 (1.60)		0.40 (0.13)		2962 (354)	
Pregnancy activity level							
Low	55 (5.2)	37.76 (2.08)	0.489	0.36 (0.11)	0.960	2764 (581)	0.005
Medium	950 (89.9)	38.02 (1.78)		0.36 (0.13)		2862 (382)	
High	32 (3.0)	37.81 (2.12)		0.36 (0.12)		2718 (399)	
Hours spent cooking over open fire during pregnancy (h/day)							
0–2	79 (7.5)	38.65 (1.64)	0.002	0.40 (0.13)	0.017	2894 (310)	0.281
2–4	531 (50.2)	37.88 (1.88)		0.35 (0.12)		2827 (414)	
4–6	447 (42.3)	38.04 (1.73)		0.36 (0.12)		2854 (388)

**Table 2:** Mean change and standard error (SE) in birth outcomes by quartile of energy and macronutrient intakes (*n* = 1057)

	Q1	Q2	Q3	Q4	p-for-trend
GA (week)					
Energy	Ref	−0.38 (0.16)*	−0.66 (0.16)*	0.13 (0.15)	0.458
Protein	Ref	−0.18 (0.12) *	−1.46 (0.15) *	− 1.99 (0.14) *	< 0.001
Fat	Ref	−0.28 (0.12)	−1.36 (0.14) *	−1.70 (0.14) *	< 0.001
Carbohydrate	Ref	0.46 (0.16) *	1.63 (0.14) *	1.91 (0.14) *	< 0.001
Fiber	Ref	−0.24 (0.14) *	−1.45 (0.15) *	−1.41 (0.15) *	< 0.001
GWG (g/week)					
Energy	Ref	−0.90 (10.80)	−13.95 (10.35)	5.85 (10.80)	0.892
Protein	Ref	−3.60 (10.80)	−49.50 (10.80) *	−71.55 (9.90) *	< 0.001
Fat	Ref	−4.50 (10.80)	−27.90 (10.80) *	−54.90 (9.90) *	< 0.001
Carbohydrate	Ref	27.00 (9.90)	75.15 (10.35) *	60.75 (9.45) *	< 0.001
Fiber	Ref	−2.25 (10.80)	−45.00 (10.35) *	−61.20 (10.35) *	< 0.001
BW (gram)					
Energy	Ref	−9.05 (34.8)	−15.0 (34.8)	77.7 (33.7) *	0.037
Protein	Ref	−26.4 (25.9) *	− 171.2 (34.7) *	− 129.0 (34.2) *	< 0.001
Fat	Ref	−62.3 (29.5) *	− 150.2 (32.5) *	−94.9 (36.6) *	< 0.001
Carbohydrate	Ref	79.8 (39.9) *	56.3 (34.4) *	128.2 (34.3) *	< 0.001
Fiber	Ref	−20.8 (29.1) *	−150.8 (33.1) *	−48.3 (35.6) *	< 0.001

Abbreviations: GA, gestational age at birth; GWG, gestational weight gain; BW, birth weight

^1^Mean energy intake 3059.3±709.2 kcal/day, mean adjusted protein intake 167.4±55.4 g/day, mean adjusted fat intake 63.2±14.4 g/day, mean adjusted carbohydrate intake 437.9±76.2 g/day, mean adjusted fiber intake 44.8±8.5 g/day. Intakes adjusted for total energy intake using the residual method

*P < 0.05, determined by analysis of variance

### Association between maternal diet, toenail as concentration and birth outcomes

Total energy was associated with GA (fully adjusted β: 0.11, 95% CI: 0.01, 0.22, *p* = 0.04), but not with GWG (fully adjusted β: 0.9, 95% CI = − 6.3, 8.1, *p* = 0.773) or BW (fully adjusted β:14.2, 95% CI:-8.9, 37.3, *p* = 0.231) (Table 3). All macronutrient intakes were significantly associated with GA and GWG (Table 3). Total energy intake was not significantly associated with ln-transformed toenail As (fully adjusted model, p = 0.77, Table 4). Protein, fat and fiber intake were positively associated with ln-transformed toenail As, while carbohydrate intake was negatively associated with ln-transformed toenail As (Table 4). Protein, fat and fiber intake were positively associated with ln-transformed toenail As, while carbohydrate intake was negatively associated with ln-transformed toenail As (Table 4). Similar to our previous findings [22], higher toenail As concentration was associated with lower GA and lower GWG, and the effect remained significant after adjusting for total energy and potential confounders (Table 5). The associations between diet, ln-transformed toenail As and birth outcomes are summarized by graphically in Additional file 1: Figure S1 to S3.

**Table 3:** Partial regression coefficient showing change in birth outcomes per increment of 1 SD^1^ of total energy and macronutrient intakes (n = 1057)

	Crude^2^		Adjusted for energy^3^		Fully Adjusted^4^	
	$\widehat{\upbeta}$(SE)	*P*-value	$\widehat{\upbeta}$(SE)	*P*-value	$\widehat{\upbeta}$(SE)	*P*-value
GA (week)						
Energy	0.12 (0.06)	0.031	–	–	0.11 (0.06)	0.040
Protein	−0.71 (0.05)	< 0.001	−0.83 (0.05)	< 0.001	− 0.87 (0.06)	< 0.001
Fat	−0.46 (0.01)	< 0.001	− 0.65 (0.05)	< 0.001	−0.58 (0.06)	< 0.001
Carbohydrate	0.57 (0.05)	< 0.001	0.79 (0.05)	< 0.001	0.79 (0.06)	< 0.001
Fiber	−0.36 (0.06)	< 0.001	−0.59 (0.05)	< 0.001	− 0.49 (0.06)	< 0.001
GWG (g/week)						
Energy	0.9 (3.6)	0.779	–	–	0.9 (3.6)	0.773
Protein	−26.6 (3.6)	< 0.001	−29.7 (3.6)	< 0.001	− 27.5 (4.5)	< 0.001
Fat	−16.2 (3.6)	< 0.001	−20.7 (3.6)	< 0.001	− 15.8 (4.1)	< 0.001
Carbohydrate	17.1 (3.6)	< 0.001	27.5 (3.6)	< 0.001	23.9 (4.5)	< 0.001
Fiber	−17.1 (3.6)	< 0.001	−23.9 (3.6)	< 0.001	−20.7 (4.1)	< 0.001
BW (gram)						
Energy	19.1 (12.2)	0.117	–	–	14.2 (11.8)	0.231
Protein	−46.0 (33.4)	< 0.001	− 58.7 (12.1)	< 0.001	− 32.3 (14.2)	0.023
Fat	−14.8 (12.2)	0.226	−32.3 (12.2)	0.008	− 11.1 (12.9)	0.389
Carbohydrate	43.5 (12.1)	< 0.001	46.9 (12.1)	< 0.001	20.2 (13.8)	0.144
Fiber	−1.45 (12.2)	0.905	−19.5 (12.2)	0.110	7.6 (13.2)	0.565

Abbreviations: SD, standard deviation; GA, gestational age at birth; GWG, gestational weight gain; BW, birth weight; SE, standard error

^1^SD for energy 606.9 kcal/day, for protein 55.6 g/day, for fat 14.2 g/day, for carbohydrates 76.1 g/day, for fiber 8.4 g/day

^2^Crude model using unadjusted intake level

^3^Intake level adjusted for energy using the residual method

^4^Intake level adjusted for energy using residual method (except for energy), regression model adjusted for BMI at the time of enrollment, exposure to secondhand tobacco smoke, betel nut chewing, age, education level, household income level, newborn sex, birth delivery location, birth delivery type, physical activity level during pregnancy, daily hours spent cooking over an open fire

**Table 4:** Partial regression coefficients showing changes in toenail arsenic concentration per increment of 1 SD of total energy and macronutrient intake^1^

Intakes	Crude^3^		Adjusted for energy^4^	Fully Adjusted^5^		
	$\widehat{\upbeta}$(SE)	*P*-value	$\widehat{\upbeta}$(SE)	*P*-value	$\widehat{\upbeta}$(SE)	*P*-value
Energy	0.01 (0.03)	0.958	–	–	−0.01 (0.03)	0.770
Protein	0.37 (0.03)	< 0.001	0.41 (0.03)	< 0.001	0.33 (0.04)	< 0.001
Fat	0.27 (0.03)	< 0.001	0.33 (0.03)	< 0.001	0.23 (0.04)	< 0.001
Carbohydrate	−0.24 (0.03)	< 0.001	− 0.40 (0.03)	< 0.001	−0.32 (0.04)	< 0.001
Fiber	0.26 (0.03)	< 0.001	0.35 (0.03)	< 0.001	0.26 (0.04)	< 0.001

Abbreviations: SD, standard deviation; SE, standard error, BMI, body mass index

^1^SD for energy 606.9 kcal/day, for protein 55.6 g/day, for fat 14.2 g/day, for carbohydrates 76.1 g/day, for fibers 8.4 g/day

^2^ln-transformed toenail arsenic concentration, unit (ln(μg/g))

^3^Crude model

^4^ Intake level and ln-transformed toenail arsenic concentration adjusted for energy using the residual method (except for energy)

^5^Intake level and ln-transformed toenail arsenic concentration adjusted for energy using residual method (except for energy), regression model adjusted for BMI at the time of enrollment, exposure to secondhand tobacco smoke, betel nut chewing, age, education level, household income level, newborn sex, birth delivery location, birth delivery type, physical activity level during pregnancy, daily hours spent cooking over an open fire

**Table 5:** Partial regression coefficient showing changes in birth outcomes per unit increase toenail arsenic^1^ (*n* = 1057)

Outcome	Crude^2^		Adjusted for energy^3^		Fully Adjusted^4^	
	$\widehat{\upbeta}$(SE)	*P*-value	$\widehat{\upbeta}$(SE)	*P*-value	$\widehat{\upbeta}$(SE)	*P*-value
*GA (week)*	−0.28 (0.01)	< 0.001	−0.28 (0.05)	< 0.001	− 0.17 (0.05)	0.001
*GWG (g/week)*	−13.1 (3.6)	< 0.001	− 13.1 (3.6)	< 0.001	−9.0 (3.6)	0.011
*BW (gram)*	−14.0 (11.2)	0.214	− 14.2 (11.2)	0.206	−5.6 (11.3)	0.618

Abbreviations: GA, gestational age at birth; GWG, gestational weight gain; BW, birth weight; SE, standard error

^1^ln-transformed toenail arsenic concentration (ln(μg/g))

### Mediation analysis considering potential exposure-mediator interaction

Mediation analyses were performed only on the pathways with significant exposure-mediator and exposure-outcome relationships. The exposure-mediator interaction was explored by including an exposure-mediator interaction term in the multiple linear regression models, significant interaction with toenail As was observed for total energy, but not for any of the macronutrients in the associations with birth outcomes. In the mediation analysis without energy adjustment, there were significant NDEs and TE by all absolute macronutrients intakes on GA and GWG, but not BW (Table 6). The directions of association were consistent with those found in the linear regression analyses. Significant NIEs by toenail As were observed for the associations between absolute fat, carbohydrate and fiber intakes on GA. The data suggested that 6% of the association between absolute fat intake and GA may be mediated by difference in toenail As level; the corresponding percent mediated was 3% for carbohydrate and 10% for fiber intake. After adjusting for total energy, the strength of association for NDEs and TEs increased, but the NIEs by toenail As were no longer statistically significant (Additional file 1: Table S1).

**Table 6:** Mediation analysis of the estimated effect^1^ (95% confidence interval, CI) of maternal energy and nutrient intake (per SD increment) on birth outcomes through toenail arsenic^2^ (ln(μg/g)) (*n* = 1057), with no energy adjustment

	Natural direct effect (95% CI)	Natural indirect effect (95% CI)	Total effect (95% CI)	Percent Mediated (%)^3^
GA (week)				
Energy^4^	0.11 (0.01, 0.20)*	0.01 (− 0.01,0.01)	0.11 (0.00, 0.22) *	
Protein	−0.66 (− 0.77, − 0.54) *	−0.01 (− 0.041, 0.02)	−0.68 (− 0.80, − 0.56) *	–
Fat	−0.34 (− 0.45, − 0.23) *	−0.02 (− 0.04, − 0.00) *	−0.36 (− 0.47, − 0.25) *	6% (1–9%)
Carbohydrate	0.47 (0.36, 0.58) *	0.02 (0.00, 0.03) *	0.49 (0.38, 0.60) *	3% (1–6%)
Fiber	−0.22 (− 0.33, − 0.10) *	−0.03 (− 0.05, − 0.01) *	−0.24 (− 0.36, − 0.13) *	10% (4–13%)
GWG (g/week)				
Protein	−21.2 (− 29.7, − 12.6) *	−1.4 (− 3.6, 0.5)	−22.5 (− 31.1, − 14.0) *	–
Fat	−9.5 (− 19.8, − 18.0) *	−1.4 (− 3.2, 0.0)	−11.3 (− 18.9, − 3.6) *	12% (0–17%)
Carbohydrate	11.7 (4.1, 19.4) *	−1.4 (− 0.5, 2.3)	12.6 (5.0, 20.3) *	–
Fiber	−11.7 (− 19.4, − 4.1) *	−1.4 (− 2.7, 0.0)	−13.1 (− 20.7, − 5.4) *	10% (0–13%)
BW (gram)				
Protein	−22.8 (−50.4, 4.8)	− 2.9 (− 3.6, 9.4)	− 19.9 (− 46.7, 6.9)	–
Carbohydrate	23.9 (− 0.79, 48.6)	− 1.3(− 4.7, 2.1)	22.6 (− 1.9, 47.0)	–

^1^The natural direct effect, natural indirect effect, and total effects reflect the change in gestational age (GA, week), gestational weight gain rate (GWG, g/week), or birth weight (BW, gram) per standard deviation (SD) increase in intake and are measured based on intake change from mean minus 1 SD to mean. Model was adjusted for BMI at the time of enrollment, exposure to environmental tobacco smoke, age, education level, household income level, newborn sex, birth delivery location, birth delivery type, physical activity level during pregnancy, and daily hours spent cooking over an open fire

^2^Absolute intake and unadjusted toenail arsenic concentration

^3^Percent mediated = (Natural indirect effect/total effect)*100%

^4^Controlled for exposure-mediator interaction

*p < 0.01

All mediation analyses had more than 95% power to detect mediation effect controlling type I error (α = 0.05). We conducted sensitivity analyses by stratifying subjects by drinking water As concentration (Additional file 1: Tables S2 to S7). The reported association remained when restricting the analysis to those exposed to water As < 50 μg/L (*n* = 833), but not for those with water As level As ≥50 μg/L (*n* = 224), possibly due to lack of power (power = 11~22%). We also stratified analysis by normal or underweight BMI; the effect estimate remained for women with normal BMI, while underweight women (BMI < 18.5 kg/m2) had a higher strength of association in general. This finding suggests that the change in diet intake had a stronger effect on birth outcomes among women with underweight BMI.

## Discussion

To our knowledge, this is the first study to use epidemiological data to study the mediating effect of long-term As exposure in the relationship of maternal diet with outcomes relating to BW. Using a prospective birth cohort in Bangladesh, where the water As level ranged widely, we were able to assess the effect of long-term As exposure on pregnancy outcomes.

Mediation analysis is an established method used in social and epidemiological studies to understand the impact of variables in causal pathways or biological mechanisms. Recently, the concept of counterfactual framework [40] has been incorporated into mediation analysis to for a more precise definition of the necessary assumption as well as for extensions to more complex model that enables exposure-mediator interaction [32]. The application of causal mediation in nutritional epidemiology generates a qualitative prospective on the NDE by exposure (diet) and the NIE by mediator (environmental exposure) while testing for interaction between exposure and mediator. Mediation analyses showed that 6% (95% CI: 1–6%) and 10% (95% CI: 4–13%) of the decreases in GA resulted from increasing absolute fat and fiber intakes, respectively, from mean minus 1 SD to the mean was mediated by increase in As exposure level associated with the change in intake. The positive associations between fat and fiber consumptions with elevated As exposure can be supported by evidence from other studies; findings from the same birth cohort indicated consumption of fish, meat and vegetable dishes were associated with higher toenail As [31]. Dark meat consumption was positively associated with toenail As in a cohort in New Hampshire, US [41]. Elevated levels of As are detectable in vegetables grown in Bangladesh [42, 43]. Further, a food survey in Pabna, Bangladesh, which collected 6 days of duplicated food samples from 47 families, showed that the median daily total As intake was 48 μg As/day from food [14]. In contrast, the negative association between carbohydrate intake and As exposure level, which drove 3% of the increase in GA, while increasing the carbohydrate intake level from the mean minus 1 SD to the mean, is less clear. Carbohydrate intake was mainly contributed by consuming grains, cereals and bread. While rice has been known to accumulate inorganic As [43, 44], our data did not show a significant association between rice consumption and elevated toenail As level [31]. Intakes of other carbohydrate-rich food, including fried bread, rice cereal and homemade snacks, were negatively associated with toenail As concentration [31]. These negative associations could indicate replacement of rice consumption with other carbohydrate sources that had lower As level, or highlight that other factors led to overall lower cumulative As exposure level.

One of the pathways [25] by which nutrition and environmental exposures may interact is the increase in overall exposure level and body burden from food intake. Diet may be a contributor to As exposure. Our data indicate a mediating effect of cumulative As level in the toenail in the association between maternal diet and birth outcomes, although the absolute magnitude of mediation was small. This mediating effect by As was observed only when looking at absolute intake of nutrients and As, but not at energy-adjusted intake.

The reason we did not observe a significant mediating effect when controlling for total energy intake could be two-fold. First, the energy-adjusted mediating effect by As exposure might be too small and the variation of macronutrient intake in proportion to total energy in our study population was not wide enough to observe a significant mediating effect. Second, there may be other potential causal pathways beyond mediation by As by which calorie-adjusted macronutrient intake and As exposure may influence GA and GWG. Examples of the potential mediators are factors that may influence one’s susceptibility to As toxicity, such as As bioavailability and As methylation capacity. Specifically, new evidence suggests that diet with a high proportion of energy from fat and protein may reduce gastrointestinal bio-accessibility of As, but may increase As speciation changes in the colon influencing As toxicity [45]. Arsenic methylation capacity, or ability to methylate, detoxify and eliminate As from the body, may be influenced by various factors, including dietary pattern and nutritional status [46]. Lower protein intake level is associated with poorer As methylation capacity [47], and consumption of seafood, seaweed and rice—which have high As content—may also interfere with the As methylation profile [46]. These factors may be changed by maternal dietary intake and alter birth outcomes. Future research to test the mediating effect of these variables will help clarify the complex relationship between maternal diet, As exposure and birth outcome.

When examining the effects of individual macronutrient intakes, we found positive associations for absolute and energy-adjusted carbohydrate intakes with GA and GWG. Carbohydrates provided a substantial proportion of the energy in the diet of our study population. Common sources of carbohydrate in the diet were rice, bread and rice cereal. A systematic review considered 18 observational studies on maternal macronutrient intake and GWG in developed settings. Three reported higher GWG associated with increased carbohydrate intake, while the other 15 did not yield consistent results [48]. Our data showed that greater protein and fat intake were associated with lower GA and GWG. Similar negative associations were reported in observational studies for protein intake, but not for fat intake [48–51]. Higher protein and fat intake may lead to a deviation from optimal dietary composition for fetal growth. High-protein diet can cause harmful effects on pregnancy: pooled data from three clinical trials with isocaloric protein supplementation found increased risks for small-for-gestational age birth (RR = 1.35, 95% CI = 1.12–1.16) [52, 53]. Higher consumption of protein may decrease GWG by requiring higher energy expenditure since the thermogenesis of protein is higher than that of carbohydrate (82, 83). The negative association could also indicate that diet is associated with increased exposure to other reproductive toxins and heavy metals. Turmeric [54] and vegetables [42] in Bangladesh contain high levels of lead, and higher fish consumption is associated with elevated mercury levels [55]. Poultry, fish and vegetables obtained from areas polluted by the tannery industry in Dhaka, Bangladesh, which is about 20 km from one of our study sites, showed unsafe levels of contamination with the heavy metals chromium, lead and mercury [56]. Greater fat intake may also increase the absorption of endocrine disrupting chemicals, which are generally lipophilic. Finally, higher consumption of protein, fat and fiber may also increase satiety, ultimately affecting the total energy intake and dietary composition. Greater fiber consumption may also impair the absorption of minerals including iron, zinc, magnesium, calcium and phosphorus [57, 58].

Our study has several strengths. First, the potential measurement error for the mediator is likely to be minimal and nondifferential. The analysis of toenail As was independent of subject collection and the research technician was blinded to subjects’ information, making the error of toenail As measurement independent of the outcome, exposure and covariates. When the mediator is continuous and the measurement error is nondifferentially misclassified, the ordinary least squares (OLS) estimator of the coefficient of the exposure-mediator regression are asymptotically unbiased [59]. Second, DCH was the primary healthcare provider in our study area; thus, all participants received the same level of prenatal care, minimizing the potential unmeasured confounding related to prenatal care. Third, we were able to control for many important potential confounders, including physical activity level during pregnancy, maternal BMI at enrollment and maternal age at the time of pregnancy, to increase the validity of our estimate. However, we recognize the potential for unmeasured confounding variables such as food quality and inter-pregnancy interval since that information was not available in the cohort.

A limitation of our study was that the FFQ does not capture the exact levels of nutritional intake. The average energy and macronutrient intake levels reported by women in our cohort were higher than both the recommended intake levels for pregnant women and the national average reported in the Bangladesh Household and Income Survey [60]. A previous analysis showed that the overestimation was non-differential to participants’ social demographic status [38]. We attempted to minimize the problem of overestimation by standardizing each intake level with its standard deviation. The problem of overestimation would not affect the validity of the FFQ to rank relative intake levels [38]; thus, provided the limited resources available to conduct dietary assessment in a large prospective study, using the FFQ was the best option to enable a good comparison of dietary intake level across study subjects. Further, an FFQ validation study using residents in Pabna, Bangladesh showed that carbohydrate intake measured by the FFQ may exhibit negative proportional bias as intake level increases, i.e., as the intake level increases, carbohydrate intake tends to be lower compared to true intake [38]. Under this circumstance, a weaker effect of association would be observed since the underestimation would bias the effect toward the null. Using the FFQ also introduces recall bias, where participants tend to report the most recent intake [61]. We conducted analyses using intake data reported by pregnant women at 28 weeks of gestation and found similar results (data not shown), suggesting the dietary pattern in our study population was consistent and the problem of recall bias should not affect the validity of the results.

## Conclusion

In our study population, maternal energy and macronutrient intakes were significantly associated with GA and GWG, and not more than 10% of the effect of each macronutrient was mediated via toenail As level. The mediating effect of As in the relationship between maternal diet and birth outcomes was small and might be due to measurement error. No significant interaction was found between maternal macronutrient intake and toenail As level in the mediation analysis. More study is needed to understand the how macronutrient intake affects As exposure level.

## Additional file

Additional file 1: **Table S1.** Associations of maternal energy and nutrient intake on birth outcomes through toenail arsenic. **Table S2.** Associations of maternal energy and nutrient intake on GA through toenail arsenic stratified by drinking water arsenic level and BMI, with no energy adjustment. **Table S3.** Associations of maternal energy and nutrient intake on GWG through toenail arsenic stratified by drinking water arsenic level and BMI, with no energy adjustment.**Table S4.** Associations of maternal energy and nutrient intake on BW through toenail arsenic stratified by drinking water arsenic level and BMI, with no energy adjustment. **Table S5.** Associations of maternal energy and nutrient intake on GA through toenail arsenic stratified by drinking water arsenic level and BMI, with energy adjustment. **Table S6.** Associations of maternal energy and nutrient intake on GWG through toenail arsenic stratified by drinking water arsenic level and BMI, with energy adjustment. **Table S7.** Associations of maternal energy and nutrient intake on BW through toenail arsenic stratified by drinking water arsenic level and BMI, with energy adjustment. **Figure S1.** Regression coefficients on the associations between maternal diet, arsenic exposure and gestational age at birth. **Figure S2.** Regression coefficients on the associations between maternal diet, arsenic exposure and gestational weight gain. **Figure S3**. Regression coefficients on the associations between maternal diet, arsenic exposure and birth weight. (DOCX 139 kb)

## Abbreviations

As: Arsenic
BMI: Body mass index
BW: Birth weight
CRM: Certified reference material
DCH: Dhaka Community Hospital
FFQ: Food Frequency Questionnaire
GA: Gestational age
GWG: Gestational weight gain
ICP-MS: Inductively coupled plasma mass spectrometry
IQR: Interquartile range
LBW: Low birth weight
LOD: Limit of detection
NDE: Natural direct effect
NIE: Natural indirect effect
SD: Standard deviation
TE: Total effect
WHO: World Health Organization
