# Supplementary material for: Mediating role of arsenic in the relationship between diet and pregnancy outcomes: prospective birth cohort in Bangladesh
Source: Environ Health. 2019 Feb 6;18:10. doi: 10.1186/s12940-019-0450-1 (PMC6364468; doi:10.1186/s12940-019-0450-1)
Supplement: Supplementary file 1 — Table S1. Associations of maternal energy and nutrient intake on birth outcomes through toenail arsenic. Table S2. Associations of maternal energy and nutrient intake on GA through toenail arsenic stratified by drinking water arsenic level and BMI, with no energy adjustment. Table S3. Associations of maternal energy and nutrient intake on GWG through toenail arsenic stratified by drinking water arsenic level and BMI, with no energy adjustment.Table S4. Associations of maternal energy and nutrient intake on BW through toenail arsenic stratified by drinking water arsenic level and BMI, with no energy adjustment. Table S5. Associations of maternal energy and nutrient intake on GA through toenail arsenic stratified by drinking water arsenic level and BMI, with energy adjustment. Table S6. Associations of maternal energy and nutrient intake on GWG through toenail arsenic stratified by drinking water arsenic level and BMI, with energy adjustment. Table S7. Associations of maternal energy and nutrient intake on BW through toenail arsenic stratified by drinking water arsenic level and BMI, with energy adjustment. Figure S1. Regression coefficients on the associations between maternal diet, arsenic exposure and gestational age at birth. Figure S2. Regression coefficients on the associations between maternal diet, arsenic exposure and gestational weight gain. Figure S3. Regression coefficients on the associations between maternal diet, arsenic exposure and birth weight. (DOCX 139 kb) [file 12940_2019_450_MOESM1_ESM.docx]

**Additional file 1**

**Table S1.** Mediation analysis of the estimated effect^1^ (95% CI) of maternal energy and nutrient intake (per SD increment) on birth outcomes through toenail arsenic^2^ (ln(μg/g)) (n=1057), with energy adjustment using the residual method

| Intake | Natural direct effect (95% CI) | Natural indirect effect (95% CI) | Total effect  (95% CI) | Percent Mediated (%)^3^ |
| --- | --- | --- | --- | --- |
| *GA (week)* | | | | |
| Protein | **-0.87 (-1.00, -0.75)** | 0.00 (-0.03, 0.04) | **-0.87 (-0.99, -0.75)** | - |
| Fat | **-0.56 (-0.67, -0.44)** | -0.02 (-0.04, 0.01) | **-0.58 (-0.69, -0.46)** | - |
| Carbohydrate | **0.79 (0.67, 0.91)** | 0.00 (-0.03, 0.03) | **0.79 (0.67, 0.91)** | - |
| Fiber | **-0.47 (-0.59, -0.65)** | -0.02 (-0.05, 0.01) | **-0.49 (-0.61, -0.37)** | - |
| *GWG (g/week)* | | | | |
| Protein | **-26.1 (-35.1, -17.1)** | -1.4 (-3.6, 1.4) | **-27.5 (-36.0, -18.5)** | - |
| Fat | **-14.0 (-22.1, -5.9)** | -1.4 (-3.2, 0.5) | **-15.8 (-23.4, -7.7)** | - |
| Carbohydrate | **22.5 (14.0, 31.1)** | -1.4 (-0.9, 3.6) | **23.9 (15.3, 32.0)** | - |
| Fiber | **-18.9 (-27.5, -10.8)** | -1.4 (-3.2, 0.5) | **-20.7 (-28.8, -12.6)** | - |
| *BW (gram)* | | | | |
| Protein | **-36.7 (-65.5, -8.0)** | 4.4 (-3.2, 12.0) | **-32.3 (-60.1, -4.5)** | - |
| Carbohydrate | 23.6 (-4.5, 51.5) | -3.4 (-10.8, 4.0) | 20.2 (-6.9, 47.2) | - |

Abbreviations: SD, standard deviation; GA, gestational age; GWG, gestational weight gain; BW, birth weight; CI, confidence interval. Bold letter indicates p<0.01

^1^ The natural direct effect, natural indirect effect, and total effects reflect the change in gestational age (week), gestational weight gain rate (g/week), or birth weight (gram) per SD increase in intake and are measured based on intake change from mean minus 1 SD to mean. Model was adjusted for BMI at the time of enrollment, exposure to environmental tobacco smoke, age, education level, household income level, newborn sex, birth delivery location, birth delivery type, physical activity level during pregnancy, and daily hours spent cooking over an open fire.

^2^ Intake level and toenail arsenic level adjusted for energy using the residual method (except for energy).

^3^ Percent mediated = (Natural indirect effect/total effect)*100%

**Table S2.** Mediation analysis of the estimated effect^1^ (95% CI) of maternal energy and nutrient intake (per SD change) on GA (week) through ln-transformed toenail arsenic [ln(μg/g)] stratified by drinking water arsenic level and BMI, with no energy adjustment

|  | **GA (week)** | | |
| --- | --- | --- | --- |
| Intake level | Natural direct effect (95% CI) | Natural indirect effect (95% CI) | Total effect  (95% CI) |
| *All subjects (N=1057)* | | | |
| Energy | **0.12 (0.01, 0.22)** | -0.00 (-0.01, 0.01) | **0.11 ( 0.01, 0.22)** |
| Protein | **-0.66 (-0.79, -0.54)** | -0.01 (-0.04, 0.02) | **-0.68 (-0.80, -0.56)** |
| Fat | **-0.34 (-0.46, -0.23)** | **-0.02 (-0.04, -0.00)** | **-0.36 (-0.47, -0.25)** |
| Carbohydrate | **0.47 (0.36, 0.58)** | **0.02 (0.00, 0.04)** | **0.49 (0.33, 0.60)** |
| Fiber | **-0.22 (-0.33, -0.10)** | **-0.03 (-0.05, -0.01)** | **-0.24 (-0.36, -0.13)** |
| *Drinking water arsenic <50* μg/L *(N=833)* | | | |
| Energy | 0.11 (-0.01, 0.22) | 0.00 (-0.01, 0.01) | 0.11 ( -0.01, 0.22) |
| Protein | **-0.68 (-0.82, -0.55)** | 0.02 (-0.00, 0.03) | **-0.68 (-0.80, -0.54)** |
| Fat | **-0.34 (-0.47, -0.21)** | 0.01 (-0.01, 0.02) | **-0.33 (-0.46, 0.02)** |
| Carbohydrate | **0.42 (0.31, 0.54)** | -0.00 (-0.00, 0.00) | **0.42 (0.30, 0.54)** |
| Fiber | **-0.19 (-0.32, - 0.07**) | 0.01 (-0.01, 0.02) | **-0.19 (-0.31, -0.06)** |
| *Drinking water arsenic* ≥50 μg/L *(N=224)* | | | |
| Energy | 0.03 (-0.31, 0.36) | 0.03 (-0.02, 0.08) | 0.05 (-0.28, 0.39) |
| Protein | **-0.25 (-0.59, -0.10)** | -0.01 (-0.05, 0.03) | **-0.26 (-0.61, -0.09)** |
| Fat | -0.14 (-0.40, 0.11) | -0.01 (-0.04, 0.02) | -0.15 (-0.41, 0.11) |
| Carbohydrate | 0.37 (-0.01, 0.83) | 0.06 (-0.04, 0.18) | 0.43 (-0.02, 0.88) |
| Fiber | -0.06 (-0.35, 0.24) | -0.00 (-0.04, 0.03) | 0.06 (-0.36, 0.24) |
| *Normal BMI (18.5≤BMI<30.0)* (*N=751*) | | | |
| Energy | 0.13 (-0.00, 0.25) | -0.00 (-0.01, 0.01) | 0.13 (-0.0, 0.25) |
| Protein | **-0.64 (-0.79, -0.50)** | -0.01 (-0.04, 0.03) | **-0.65 (-0.79, -0.51)** |
| Fat | **-0.33 (-0.47, -0.20)** | -0.02 (-0.04, 0.01) | **-0.35 (-0.48, -0.22)** |
| Carbohydrate | **0.47 (0.34, 0.60)** | 0.01 (-0.01, 0.03) | **0.48 (0.35, 0.61)** |
| Fiber | **-0.23 (-0.36, -0.09)** | -0.02 (-0.04, 0.01) | **-0.24 (-0.38, -0.11)** |
| *Underweight women (BMI<18.5) (N=296)* | | | |
| Energy | 0.02 (-0.20, 0.23) | 0.00 (-0.03, 0.04) | 0.02 (-0.20, 0.24) |
| Protein | **-0.72 (-0.95, -0.49)** | -0.04 (-0.01, 0.02) | **-0.76 (-0.98, -0.54)** |
| Fat | **-0.38 (-0.60, -0.16)** | -0.04 (-0.09, 0.01) | **-0.42 (-0.64, -0.20)** |
| Carbohydrate | **0.41 (0.18, 0.64)** | 0.04 (-0.00, 0.09) | **0.46 (0.23, 0.68)** |
| Fiber | -0.22 (-0.45, 0.02) | -0.05 (-0.11, 0.01) | **-0.27 (-0.50, -0.03)** |

Abbreviations: SD, standard deviation; GA, gestational age; BMI, body mass index; CI, confidence interval. Bold letter indicates p<0.01

^1^ The natural direct effect, natural indirect effect, and total effects reflect the change in gestational age at birth (week), gestational weight gain (g/week), or birth weight (gram) per SD increase in intake and are measured based on intake change from mean minus 1 SD to mean. Model was adjusted for BMI at the time of enrollment, exposure to environmental tobacco smoke, age, education level, household income level, newborn sex, birth delivery location, birth delivery type, physical activity level during pregnancy, and daily hours spent cooking over open fire.

**Table S3.** Mediation analysis of the estimated effect^1^ (95% CI) of maternal energy and nutrient intake (per SD change) on GWG (g/week) through ln-transformed toenail arsenic [ln(μg/g)] stratified by drinking water arsenic level and BMI, with no energy adjustment

|  | **GWG (g/week)** | | | |
| --- | --- | --- | --- | --- |
| Intake level | Natural direct effect (95% CI) | Natural indirect effect (95% CI) | Total effect  (95% CI) | |
| *All subjects (N=1057)* | | | | |
| Energy | 1.4 (-6.3, 8.6) | 0.5 (-0.5, 0.5) | 0.9 (-6.3, 8.6) | |
| Protein | **-21.2 (-29.7, -12.6)** | -1.4 (-3.6, 0.5) | **-22.5 (-31.1, -14.0)** | |
| Fat | **-9.5 (-19.8, -18.0)** | -1.4 (-3.2, 0.0) | **-11.3 (-18.9, -3.6)** | |
| Carbohydrate | **11.7 (4.1, 19.4)** | 1.4 (-0.5, 2.3) | **12.6 (5.0, 20.3)** | |
| Fiber | **-11.7 (-19.4, -4.1)** | -1.4 (-2.7, 0.0) | **-13.1 (-20.7, -5.4)** | |
| *Drinking water arsenic <50* μg/L *(N=833)* | | | | |
| Energy | 0.5 (-7.7, 8.1) | 0.0 (-0.9, 0.5) | 0.5 (-8.1, 8.1) | |
| Protein | **-20.7 (-30.6, -10.8)** | -0.5 (-1.4, 0.9) | **-21.2 (-31.1, -11.3)** | |
| Fat | -8.6 (-18.0, 0.5) | -0.5 (-1.4, 0.5) | -9.0 (-18.5, 1.4) | |
| Carbohydrate | **9.0 (1.8, 17.6)** | 0.5 (-0.5, 0.5) | **9.0 (0.5, 17.6)** | |
| Fiber | **-13.5 (-22.5, -0.5)** | -0.5 (-1.4, 0.5) | **-14.0 (-23.0, -4.5)** | |
| *Drinking water arsenic* ≥50 μg/L *(N=224)* | | | | |
| Energy | -7.2 (-25.7, 11.7) | 0.5 (-2.3, 2.3) | -7.2 (-25.7, 11.3) | |
| Protein | -8.6 (-28.4, 10.8) | 0.5 (-0.9, 1.4) | -9.0 (-27.9, 10.8) | |
| Fat | -9.9 (-24.8, 4.5) | 0.9 (-0.5, 0.9) | -9.9 (-24.8, 4.5) | |
| Carbohydrate | -0.9 (-27.0, 25.2) | 0.5 (-7.2, 5.9) | -1.8 (-27.0, 23.9) | |
| Fiber | -4.5 (-21.2, 12.2) | -4.5 (-0.5, 0.5) | -0.5 (-22.5, 12.2) | |
| *Normal BMI (18.5≤BMI<30.0)* (*N=751*) | | | |  |
| Energy | 1.4 (-7.2, 9.5) | -0.5 (-7.2, 0.5) | 0.9 (-7.2, 9.5) |  |
| Protein | **-18.0 (-27.9, -7.7)** | -1.4 (-27.9, 0.9) | **-19.4 (-29.3, -9.5)** |  |
| Fat | -5.4 (-14.4, 3.6) | -1.4 (-14.4, 0.5) | -6.8 (-15.8, 2.3) |  |
| Carbohydrate | 9.0 (-0.5, 18.0) | 0.9 (-0.5, 2.3) | **9.9 (0.9, 18.9)** |  |
| Fiber | **-9.0 (-18.0, -0.5)** | -1.4 (-18.0, 0.5) | **-10.4 (-18.9, 1.4)** |  |
| *Underweight women (BMI<18.5) (N=296)* | | | |  |
| Energy | -1.4 (-16.7, 13.5) | 0.5 (-16.7, 0.5) | 1.4 (-16.7, 14.0) |  |
| Protein | **-26.1 (-43.7, -9.5)** | -2.3 (-43.7, 2.3) | **-28.4 (-45.0, -12.2)** |  |
| Fat | **-19.8 (-35.6, -3.6)** | -1.8 (-35.6, 0.9) | **-22.5 (-36.9, -5.9)** |  |
| Carbohydrate | 14.9 (-1.8, 31.5) | 2.3 (-1.8, 5.4) | **17.1 (0.5, 33.3)** |  |
| Fiber | **-21.6 (-38.3, -4.5)** | -2.3 (-38.3, 1.4) | **-23.9 (-40.1, -7.2)** |  |

Abbreviations: SD, standard deviation; GWG, gestational weigh gain; BMI, body mass index; CI, confidence interval. Bold letter indicates p<0.01

^1^ The natural direct effect, natural indirect effect, and total effects reflect the change in gestational age at birth (week), gestational weight gain (g/week), or birth weight (gram) per SD increase in intake and are measured based on intake change from mean minus 1 SD to mean. Model was adjusted for BMI at the time of enrollment, exposure to environmental tobacco smoke, age, education level, household income level, newborn sex, birth delivery location, birth delivery type, physical activity level during pregnancy, and daily hours spent cooking over an open fire.

**Table S4.** Mediation analysis of the estimated effect^1^ (95% CI) of maternal energy and nutrient intake (per SD change) on BW (gram) through ln-transformed toenail arsenic [ln(μg/g)] stratified by drinking water arsenic level and BMI, with no energy adjustment

|  | **BW (gram)** | | | |
| --- | --- | --- | --- | --- |
| Intake level | Natural direct effect (95% CI) | Natural indirect effect (95% CI) | Total effect  (95% CI) | |
| *All subjects (N=1057)* | | | | |
| Energy | 14.2 ( -9.1, 37.4) | 0.1 (-0.4, 0.43) | 14.2 (-9.1, 37.4) | |
| Protein | -22.8 (-50.4, 4.8) | 2.9 (-3.6, 9.4) | -19.9 (-46.7, 6.9) | |
| Fat | -0.1 (-24.8, 24.6) | 1.7 (-3.1, 5.1) | 0.9 (-23.5, 25.3) | |
| Carbohydrate | 23.9 (-0.8, 48.6) | -1.3 (-4.8, 2.1) | 22.6 (-1.9, 47.0) | |
| Fiber | 15.4 (-9.5, 40.4) | 0.6 (-3.3, 4.6) | 16.1 ( -8.6, 40.7) | |
| *Drinking water arsenic <50* μg/L *(N=833)* | | | | |
| Energy | 7.1 (-16.4, 30.5) | 0.6 (-0.9, 2.2) | 7.7 (-15.7, 31.1) | |
| Protein | **-36.6 (-65.7, -7.4)** | 2.5 (-1.5, 6.5) | **-34.1 (-36.1, -5.2)** | |
| Fat | -11.4 (-38.4, 15.6) | 1.5 (-1.5, 4.4) | -9.9 (-36.8, 16.9) | |
| Carbohydrate | 21.4 (-3.3, 46.1) | -0.1 (-0.1, 0.8) | 21.3 (-3.5, 46.0) | |
| Fiber | 4.4 (-22.2, 31.0) | 1.3 (-1.6, 4.2) | 5.7 (-20.8, 32.1) | |
| *Drinking water arsenic* ≥50 μg/L *(N=224)* | | | | |
| Energy | 48.6 (-34.0, 131.1) | -0.7 (-10.5, 9.2) | 47.9 (-34.1, 129.9) | |
| Protein | 55.2 (-31.2, 141.5) | -0.2 ( -5.2, 4.8) | 55.0 (-31.2, 141.2) | |
| Fat | 51.6 (-13.1, 116.3) | -0.2 (-3.7, 3.4) | 51.4 (-13.2, 116.0) | |
| Carbohydrate | 5.8 (-110.6, 112.1) | -0.9 (-29.6, 29.1) | 5.5 (-107.1, 118.1) | |
| Fiber | **75.5 (2.0, 149.1)** | -0.3 (-1.4, 1.3) | **75.5 (2.0, 149.0)** | |
| *Normal BMI (18.5≤BMI<30.0)* (*N=751*) | | | |  |
| Energy | 21.4 (-5.7, 48.6) | 0.1 (-0.7, 1.0) | 21.6 (-5.5, 48.7) |  |
| Protein | -6.9 (-39.8, 26.0) | 3.3 (-4.2, 10.8) | -3.6 (-35.6, 28.6) |  |
| Fat | 13.5 (-15.7, 42.6) | 1.6 (-3.3, 6.4) | 15.0 (-13.7, 43.8) |  |
| Carbohydrate | 23.9 (-4.9, 52.6) | -1.8 (-5.5, 1.9) | 22.1 (-6.4, 50.6) |  |
| Fiber | 25.9 (-2.8, 54.6) | 1.2 (-3.2, 5.5) | 27.7 (-1.37, 55.5) |  |
| *Underweight women (BMI<18.5) (N=296)* | | | |  |
| Energy | -14.6 (-61.0, 31.9) | 0.2 (-1.6, 2.0) | -14.4 (-60.9, 32.1) |  |
| Protein | **-64.6 (-117.1, -12.1)** | 0.0 (-12.8, 12.8) | **-64.6 (-115.5, -13.7)** |  |
| Fat | -36.9 (-85.5, 11.6) | -1.4 (-9.3, 6.5) | -38.6 (-86.3, 9.5) |  |
| Carbohydrate | 14.9 (-36.4, 66.3) | 2.3 (-6.7, 11.4) | 17.2 (-33.4, 67.8) |  |
| Fiber | -30.8 (-82.5, 21.0) | -1.8 (-11.2, 7.6) | -32.6 (-83.5, 18.3) |  |

Abbreviations: SD, standard deviation; BW, birth weight; BMI, body mass index; CI, confidence interval. Bold letter indicates p<0.01

^1^ The natural direct effect, natural indirect effect, and total effects reflect the change in gestational age at birth (week), gestational weight gain (g/week), or birth weight (gram) per SD increase in intake and are measured based on intake change from mean minus 1 SD to mean. Model was adjusted for BMI at the time of enrollment, exposure to environmental tobacco smoke, age, education level, household income level, newborn sex, birth delivery location, birth delivery type, physical activity level during pregnancy, and daily hours spent cooking over an open fire.

**Table S5.** Mediation analysis of the estimated effect^1^ (95% CI) of maternal energy and nutrient intake (per SD change) on GA (week) through ln-transformed toenail arsenic [ln(μg/g)] stratified by drinking water arsenic level and BMI, with energy adjustment^2^

|  | **GA (week)** | | |
| --- | --- | --- | --- |
| Intake level | Natural direct effect (95% CI) | Natural indirect effect (95% CI) | Total effect  (95% CI) |
| *All subjects (N=1057)* | | | |
| Protein | **-0.87 (-1.00, -0.75)** | 0.00 (-0.03, 0.04) | **-0.87 (-0.99, -0.75)** |
| Fat | **-0.56 (-0.67, -0.44)** | -0.02 (-0.04, 0.01) | **-0.58 (-0.69, -0.46)** |
| Carbohydrate | **0.79 (0.66, 0.91)** | 0.00 (-0.03, 0.03) | **0.79 (0.67, 0.91)** |
| Fiber | **-0.47 (-0.59, -0.65)** | -0.02 (-0.05, 0.01) | **-0.49 (-0.61, -0.37)** |
| *Drinking water arsenic <50* μg/L *(N=833)* | | | |
| Protein | **-0.89 (-1.03, -0.76)** | 0.02 (-0.00, 0.03) | **-0.88 (-1.01, -0.74)** |
| Fat | **-0.56 (-0.68, -0.42)** | 0.01 (-0.01, 0.02) | **-0.55 (-0.68, -0.42)** |
| Carbohydrate | **0.79 (0.66, 0.93)** | -0.01 (-0.03, 0.01) | **0.78 (0.65, 0.91)** |
| Fiber | **-0.45 (-0.58, -0.31)** | 0.07 (-0.00, 0.01) | **-0.44 (-0.58, -0.31)** |
| *Drinking water arsenic* ≥50 μg/L *(N=224)* | | | |
| Protein | -0.41 (-0.83, 0.00) | 0.03 (-0.10, 0.03) | **-0.46 (-0.86, -0.03)** |
| Fat | -0.26 (-0.57, 0.04) | -0.03 (-0.08, 0.03) | -0.29 (-0.60, 0.10) |
| Carbohydrate | 0.39 (-0.00, 0.77) | 0.04 (-0.03, 0.11) | **0.42 (0.04, 0.80)** |
| Fiber | -0.12 (-0.44, 0.21) | -0.03 (-0.08, 0.02) | -0.15 (-0.47, 0.18) |
| *Normal BMI (18.5≤BMI<30.0)*  (n=751) | | | |
| Protein | **-0.86 (-1.01, -0.71)** | -0.01 (-0.03, 0.05) | **-0.85 (-0.99, -0.70)** |
| Fat | **-0.55 (-0.69, -0.42)** | -0.01 (-0.04, 0.02) | **-0.56 (-0.70, -0.43)** |
| Carbohydrate | **0.78 (0.63, 0.92)** | -0.01 (-0.04, 0.03) | **0.77 (0.63, 0.91)** |
| Fiber | **-0.48 (-0.62, -0.34)** | -0.01 (-0.04, 0.02) | **-0.49 (-0.62, -0.36)** |
| *Underweight women (BMI<18.5)(n=296)* | | | |
| Protein | **-0.93 (-1.17, -0.69)** | -0.00 (-0.09, 0.05) | **-0.95 (-1.18, -0.72)** |
| Fat | **-0.55 (-0.79, -0.32)** | -0.05 (-0.10, 0.01) | **-0.60 (-0.83, -0.37)** |
| Carbohydrate | **0.81 (0.56, 1.05)** | 0.03 (-0.04, 0.10) | **0.84 (0.61, 1.08)** |
| Fiber | **-0.40 (-0.67, -0.13)** | -0.07 (-0.15, 0.01) | **-0.47 (-0.73, -0.21)** |

Abbreviations: SD, standard deviation; GA, gestational age at birth; BMI, body mass index; CI, confidence interval. Bold letter indicates p<0.01

^1^ The natural direct effect, natural indirect effect, and total effects reflect the change in gestational age at birth (week), gestational weight gain (g/week), or birth weight (gram) per SD increase in intake and are measured based on intake change from mean minus 1 SD to mean. Model was adjusted for BMI at the time of enrollment, exposure to environmental tobacco smoke, age, education level, household income level, newborn sex, birth delivery location, birth delivery type, physical activity level during pregnancy, and daily hours spent cooking over an open fire.

^2^ Intake level and toenail arsenic level adjusted for energy using the residual method (except for energy).

**Table S6.** Mediation analysis of the estimated effect^1^ (95% CI) of maternal energy and nutrient intake (per SD change) on GWG (g/week) through ln-transformed toenail arsenic [ln(μg/g)] stratified by drinking water arsenic level and BMI, with energy adjustment^2^

|  | **GWG (g/week)** | | | |
| --- | --- | --- | --- | --- |
| Intake level | Natural direct effect (95% CI) | Natural indirect effect (95% CI) | Total effect  (95% CI) | |
| *All subjects (N=1057)* | | | | |
| Protein | **-26.1 (-35.1, -17.1)** | -1.4 (-3.6, 1.4) | **-27.5 (-36.0, -18.5)** | |
| Fat | **-14.0 (-22.1, -5.9)** | -1.4 (-3.2, 0.5) | **-15.8 (-23.4, -7.7)** | |
| Carbohydrate | **22.5 (14.0, 31.1)** | 1.4 (-0.9, 3.6) | **23.9 (15.3, 32.0)** | |
| Fiber | **-18.9 (-27.5, -10.8)** | -1.4 (-3.2, 0.5) | **-20.7 (-28.8, -12.6)** | |
| *Drinking water arsenic <50* μg/L *(N=833)* | | | | |
| Protein | **-25.2 (-35.6, -14.9)** | -0.5 (-1.4, 0.9) | **-25.2 (-35.6, -14.9)** | |
| Fat | **-11.7 (-21.2, -2.3)** | -0.5 (-1.4, 0.5) | **-12.2 (-21.6, -2.7)** | |
| Carbohydrate | **21.2 (11.3, 31.5)** | -0.5 (-0.9, 1.4) | **21.6 (11.3, 31.5)** | |
| Fiber | **-21.6 (-31.5, -11.7)** | -0.5 (-1.4, 0.5) | **-22.1 (-31.5, -12.2)** | |
| *Drinking water arsenic* ≥50 μg/L *(N=224)* | | | | |
| Protein | -9.0 (-32.4, 14.4) | 0.5 (-2.7, 3.6) | -8.6 (-32.0, 14.4) | |
| Fat | -13.1 (-30.2, 4.1) | 0.5 (-2.3, 3.2) | -12.6 (-29.7, 4.5) | |
| Carbohydrate | 11.7 (-9.9, 33.3) | -0.9 (-4.5, 3.2) | 10.8 (-10.8, 32.4) | |
| Fiber | -0.9 (-19.8, 17.1) | -0.5 (-2.3, 2.7) | -0.9 (-19.4, 17.1) | |
| *Normal BMI (18.5≤BMI<30.0)*  (*N=751*) | | | |  |
| Protein | **-22.1 (-32.9, -11.7)** | -1.4 (-4.1, 4.1) | **-23.4 (-33.8, -13.1)** |  |
| Fat | **-8.1 (-17.6, -1.4)** | -1.8 (-3.6, 0.5) | **-9.9 (-18.9, -0.5)** |  |
| Carbohydrate | **17.1 (6.8, 27.5)** | 1.4 (-0.9, 4.1) | **18.9 (8.6, 28.8)** |  |
| Fiber | **-14.9 (-24.3, -5.4)** | -1.4 (-3.2, 0.5) | **-16.2 (-25.2, -6.8)** |  |
| *Underweight women (BMI<18.5) (N=296)* | | | |  |
| Protein | **-32.4 (-50.9, -14.4)** | -1.8 (-7.2, 3.6) | **-34.7 (-52.2, -17.1)** |  |
| Fat | **-27.0 (-43.7, -9.9)** | -2.3 (-5.9, 1.8) | **-28.8 (-45.5, -12.2)** |  |
| Carbohydrate | **33.3 (15.3, 51.8)** | 1.8 (-3.6, 7.2) | **35.1 (18.0, 52.7)** |  |
| Fiber | **-35.1 (-54.0, -16.2)** | -1.8 (-7.2, 3.6) | **-36.9 (-55.4, -18.9)** |  |

Abbreviations: SD, standard deviation; GWG, gestational weight gain; BMI, body mass index; CI, confidence interval. Bold letter indicates p<0.01

^1^ The natural direct effect, natural indirect effect, and total effects reflect the change in gestational age at birth (week), gestational weight gain (g/week), or birth weight (gram) per SD increase in intake and are measured based on intake change from mean minus 1 SD to mean. Model was adjusted for BMI at the time of enrollment, exposure to environmental tobacco smoke, age, education level, household income level, newborn sex, birth delivery location, birth delivery type, physical activity level during pregnancy, and daily hours spent cooking over an open fire.^2^ Intake level and toenail arsenic level adjusted for energy using the residual method (except for energy)

**Table S7.** Mediation analysis of the estimated effect^1^ (95% CI) of maternal energy and nutrient intake (per SD change) on BW (gram) through ln-transformed toenail arsenic [ln(μg/g)] stratified by drinking water arsenic level and BMI, with energy adjustment^2^

|  | **GW (gram)** | | | |
| --- | --- | --- | --- | --- |
| Intake level | Natural direct effect (95% CI) | Natural indirect effect (95% CI) | Total effect  (95% CI) | |
| *All subjects (N=1057)* | | | | |
| Protein | **-36.7 (-65.5, -8.0)** | 4.4 (-3.23 12.0) | **-32.3 (-60.1, -4.5)** | |
| Fat | -12.9 (-38.7, 12.8) | 1.8 (-3.4, 7.1) | -11.1 (-36.3, 14.1) | |
| Carbohydrate | 23.6 (-4.5, 51.5) | -3.4 (-10.8, 4.0) | 20.2 (-6.9, 47.3) | |
| Fiber | 6.5 (-20.1, 33.1) | 1.1 (-4.7, 7.0) | 7.6 (-18.3, 33.6) | |
| *Drinking water arsenic <50* μg/L *(N=833)* | | | | |
| Protein | **-48.8 (-79.2, -18.5)** | 2.4 (-1.4, 6.2) | **-46.4 (-76.6, -16.3)** | |
| Fat | -22.2 (-50.0, 5.6) | 1.3 (-1.3, 3.8) | -21.0 (-48.6, 6.7) | |
| Carbohydrate | **36.7 (6.8, 66.5)** | -2.1 (-5.7, 1.5) | **34.6 (4.9, 64.2)** | |
| Fiber | -2.8 (-31.6, 26.0) | 1.2 (-1.5, 3.9) | -1.6 (-30.3, 27.1) | |
| *Drinking water arsenic* ≥50 μg/L *(N=224)* | | | | |
| Protein | 54.6 (-49.3, 158.6) | -1.1 (-15.0, 12.8) | 53.5 (-49.6, 156.6) | |
| Fat | 58.7 (-18.2, 135.6) | -1.5 (-13.3, 10.3) | 57.2 (-18.8, 133.2) | |
| Carbohydrate | -83.3 (-180.0, 13.5) | 2.5 (-13.6, 18.5) | -80.8 (-176.3, 14.7) | |
| Fiber | 79.9 (-1.4, 161.1) | -1.5 (-12.2, 9.2) | 78.4 (-2.1, 158.9) | |
| *Normal BMI (18.5≤BMI<30.0)* (*N=751*) | | | |  |
| Protein | -22.9 (-57.2, 11.5) | 4.7 (-4.0 13.4) | -18.1 (-51.4, 15.2) |  |
| Fat | -1.6 (-31.8, 28.6) | 2.4 (-3.6, 8.4) | 0.8 (-28.8, 30.4) |  |
| Carbohydrate | 9.9 (-23.2, 43.0) | -3.8 (-12.1, 4.5) | 6.1 (-26.0, 38.2) |  |
| Fiber | 14.0 (-16.2, 44.2) | 1.8 (-4.2, 7.7) | 15.8 (-13.9, 45.4) |  |
| *Underweight women (BMI<18.5) (N=296)* | | | |  |
| Protein | **-73.1 (-129.7, -16.6)** | 1.4 (15.5, 17.8) | **-71.8 (-126.0, -17.6)** |  |
| Fat | -39.4 (-91.8, 12.9) | -1.5 (-13.2, 10.2) | -40.9 (-92.0, 10.1) |  |
| Carbohydrate | **59.7 (3.3, 116.2)** | -0.3 (-6.7, 16.1) | **59.4 (5.4, 113.4)** |  |
| Fiber | -32.0 (-90.8, 26.9) | -2.3 (-90, 14.4) | -34.2 (-90.7, 22.2) |  |

Abbreviations: SD, standard deviation; BW, birth weight; BMI, body mass index; CI, confidence interval. Bold letter indicates p<0.01

^1^ The natural direct effect, natural indirect effect, and total effects reflect the change in gestational age at birth (week), gestational weight gain (g/week), or birth weight (gram) per SD increase in intake and are measured based on intake change from mean minus 1 SD to mean. Model was adjusted for BMI at the time of enrollment, exposure to environmental tobacco smoke, age, education level, household income level, newborn sex, birth delivery location, birth delivery type, physical activity level during pregnancy, and daily hours spent cooking over an open fire.

^2^ Intake level and toenail arsenic level adjusted for energy using the residual method (except for energy).

(a)

Total Energy Intake

Toenail [As]

GA

-0.01 (0.03)

-0.173 (0.052)*

0.11 (0.06)*

(b)

Protein Intake

Toenail [As]

GA

0.33 (0.04)*

-0.17 (0.05)*

-0.87 (0.06)*

(c)

Fat Intake

Toenail [As]

GA

0.23 (0.04)*

-0.17 (0.05)*

-0.58 (0.06)*

(d)

Carbohydrate Intake

Toenail [As]

GA

-0.32 (0.04)*

-0.17 (0.05)*

0.79 (0.06)*

(e)

Fiber Intake

Toenail [As]

GA

0.26 (0.04)*

-0.17 (0.05)*

-0.49 (0.06)*

**Figure S1.** Partial regression coefficients (and standard errors) on the associations between maternal diet, arsenic exposure and gestational age at birth (GA), controlling for potential confounders with energy adjustment. *p<0.05

(a)

Total Energy Intake

Toenail [As]

GWG

-0.01 (0.03)

-9.0 (3.6)*

9.0 (3.6)

(b)

Protein Intake

Toenail [As]

GWG

0.33 (0.04)*

-9.0 (3.6)*

-27.5 (4.5)*

)*

(c)

Fat Intake

Toenail [As]

GWG

0.23 (0.04)*

-9.0 (3.6)*

-15.8 (4.1)*

(d)

Carbohydrate Intake

Toenail [As]

GWG

-0.32 (0.04)*

-9.0 (3.6)*

23.9 (4.5)*

(e)

Fiber Intake

Toenail [As]

GWG

0.26 (0.04)*

-9.0 (3.6)*

-20.7 (4.1)*

**Figure S2**. Partial regression coefficients (and standard errors) on the associations between maternal diet, arsenic exposure and gestational weight gain (GWG), controlling for potential confounders with energy-adjustment. *p<0.05

(a)

Total Energy Intake

Toenail [As]

BW

-0.01 (0.03)

-14.2 (11.2)

19.1 (12.2)

(b)

Protein Intake

Toenail [As]

BW

0.33 (0.04)*

-14.2 (11.2)

-58.7 (12.1)*

(c)

Fat Intake

Toenail [As]

BW

0.23 (0.04)*

-14.2 (11.2)

-32.3 (12.2)*

(d)

Carbohydrate Intake

Toenail [As]

BW

-0.32 (0.04)*

-14.2 (11.2)

46.9 (12.1)*

(e)

Fiber Intake

Toenail [As]

BW

0.26 (0.04)*

-14.2 (11.2)

-19.5 (12.2)

**Figure S3**. Partial regression coefficients (and standard errors) on the associations between maternal diet, arsenic exposure and birth weight (BW), controlling for potential confounders with energy-adjustment. *p<0.05
